# TOWARD OPTIMIZED CLINICAL TRIALS OF BOTANICAL DIETARY SUPPLEMENTS IN OLDER ADULTS

**DOI:** 10.1093/geroni/igae098.2843

**Published:** 2024-12-31

**Authors:** Alex Speers, Amala Soumyanath

**Affiliations:** Oregon Health & Science University, Portland, Oregon, United States; Oregon Health & Science University, Portland, Oregon, United States

## Abstract

The use of dietary supplements, which includes botanical dietary supplements, is increasingly common among older adults. This is concerning due to a) the lack of high-quality efficacy studies for botanical products, b) issues with product quality and variability, and c) the potential for herb-drug interactions, which is increased in older adults due to higher rates of polypharmacy. Given the growing popularity of these products among consumers, more rigorous data are needed, but a rational, stepwise approach is required. At Oregon Health & Science University (OHSU) and collaborating institutes, a pipeline is being developed to study botanical dietary supplements in an older population. The BENFRA Botanical Dietary Supplements Center at OHSU studies Botanicals Enhancing Neurological and Functional Resilience in Aging, with a focus on two botanicals: Centella asiatica (gotu kola) and Withania somifera (ashwagandha). This work includes product characterization studies, exploration of mechanisms in vitro and in vivo, and the development of methods for measuring plant compounds in plasma. Separate from the BENFRA Center, studies are now underway at OHSU to investigate the safety, pharmacokinetics, and biological signatures of these botanicals in humans. Collectively, this pipeline of research will generate the necessary data to support future optimized efficacy trials in older adults. The presentation will outline the rigorous studies being undertaken and provide preliminary data from these preclinical and clinical investigations.

